# Post-Transplant Cyclophosphamide and Tacrolimus—Mycophenolate Mofetil Combination Governs GVHD and Immunosuppression Need, Reducing Late Toxicities in Allogeneic Peripheral Blood Hematopoietic Cell Transplantation from HLA-Matched Donors

**DOI:** 10.3390/jcm10061173

**Published:** 2021-03-11

**Authors:** Fabrizio Carnevale-Schianca, Daniela Caravelli, Susanna Gallo, Paolo Becco, Luca Paruzzo, Stefano Poletto, Alessandra Polo, Monica Mangioni, Milena Salierno, Massimo Berger, Rosanna Pessolano, Francesco Saglio, Daniela Gottardi, Delia Rota-Scalabrini, Giovanni Grignani, Marco Fizzotti, Ivana Ferrero, Pio Manlio Mirko Frascione, Lorenzo D’Ambrosio, Valentina Gaidano, Loretta Gammaitoni, Dario Sangiolo, Andrea Saglietto, Elena Vassallo, Alessandro Cignetti, Massimo Aglietta, Franca Fagioli

**Affiliations:** 1 Department of Medical Oncology, Candiolo Cancer Institute, FPO-IRCCS, 10060 Candiolo, Italy; daniela.caravelli@ircc.it (D.C.); paolo.becco@aslbi.piemonte.it (P.B.); delia.rotascalabrini@ircc.it (D.R.-S.); giovanni.grignani@ircc.it (G.G.); marco.fizzotti@ircc.it (M.F.); lorenzo.dambrosio.md@gmail.com (L.D.); loretta.gammaitoni@ircc.it (L.G.); dario.sangiolo@ircc.it (D.S.); 2 Turin Metropolitan Transplant Center, Hematopoietic Stem Cells Unit, Department of Medical Oncology, Candiolo Cancer Institute, FPO-IRCCS, 10060 Candiolo, Italy; luca.paruzzo@ircc.it (L.P.); stefano.poletto@ircc.it (S.P.); piomanliomirko.frascione@ircc.it (P.M.M.F.); 3 Department of Hematology and Cell Therapy, University of Turin, A.O. Ordine Mauriziano, 10128 Turin, Italy; susygallo2014@gmail.com (S.G.); dgottardi@mauriziano.it (D.G.); vale_gaidano@hotmail.com (V.G.); acignetti@mauriziano.it (A.C.); 4 Department of Oncology, University of Turin, 10060 Candiolo, Italy; 5 Collection and Processing Laboratory Candiolo, Cancer Institute, FPO-IRCCS, 10060 Candiolo, Italy; alessandra.polo@ircc.it (A.P.); monica.mangioni@ircc.it (M.M.); milena.salierno@ircc.it (M.S.); 6 Pediatric Onco–Hematology, Stem Cell Transplantation, and Cellular Therapy Division, Turin Metropolitan Transplant Center, A.O.U. Citta’della Salute e della Scienza di Torino, Ospedale Infantile Regina Margherita, 10126 Turin, Italy; massimoberger@gmail.com (M.B.); rosanna.pessolano@gmail.com (R.P.); francesco.saglio@unito.it (F.S.); Ivana.Ferrero@unito.it (I.F.); elena.vassallo@unito.it (E.V.); franca.fagioli@unito.it (F.F.); 7 Cardiology Division, A.O.U. Citta’della Salute e della Scienza di Torino, Department of Medical Sciences, University of Turin, 10126 Turin, Italy; andrea.saglietto@live.com; 8 Department of Public Health and Pediatrics, University of Turin, 10126 Turin, Italy

**Keywords:** allogeneic hematopoietic cell transplantation, graft-versus-host disease, post-transplant cyclophosphamide, immunosuppression modulation, long term outcomes

## Abstract

Combined direct antineoplastic activity and the long-lasting immunological effects of allogeneic hematopoietic cell transplant (HCT) can cure many hematological malignancies, but broad adoption requires non-relapse mortality (NRM) rates and graft-versus-host disease (GVHD) control. Recently, posttransplant cyclophosphamide (PTCy) given after a bone marrow transplant significantly reduced GVHD-incidence, while PTCy given with tacrolimus/mofetil mycophenolate (T/MMF) showed activity following allogeneic peripheral blood stem cell transplantation (alloPBSCT). Here, we report the experience of a larger cohort (85 consecutive patients) and expanded follow-up period (03/2011–12/2019) with high-risk hematological malignancies who received alloPBSCT from Human-Leukocyte-Antigens HLA-matched unrelated/related donors. GVHD-prophylaxis was PTCy 50 mg/kg (days+3 and +4) combined with T/MMF (day+5 forward). All patients stopped MMF on day+28 with day+110 = median tacrolimus discontinuation. Cumulative incidences were 12% for acute and 7% for chronic GVHD- and no GVHD-attributed deaths. For surviving patients, the 12, 24, and 36-month probabilities of being off immunosuppression were 92, 96, and 96%, respectively. After a 36-month median follow-up, NRM was 4%; median event-free survival (EFS) and overall survival (OS) had yet to occur. One- and two-year chronic GVHD-EFS results were 57% (95% CI, 46–68%) and 53% (95% CI, 45–61%), respectively, with limited late infections and long-term organ toxicities. Disease relapse caused the most treatment failures (38% at 2 years), but low transplant toxicity allowed many patients (14/37, 38%) to receive donor lymphocyte infusions as a post-relapse strategy. We confirmed that PTCy+T/MMF treatment effectively prevented acute and chronic GVHD and limited NRM to unprecedented low rates without loss of disease control efficacy in an expanded patient cohort. This trial is registered at U.S. National Library of Medicine as #NCT02300571.

## 1. Introduction

In recent years, extraordinary advances have been made in therapeutics for many hematological malignancies; however, allogeneic hematopoietic cell transplant (HCT) represents the only potentially curative and globally affordable treatment for most of them [1,2,3,4,5,6,7].

To allow the widespread use of HCT, issues of transplant toxicity and disease relapse require immediate address [2,3,5,6,7]. The key to both of these issues appears to be graft-versus-host disease (GVHD). As the principal cause of death in allogeneic HCT, GVHD prevention or treatment requires immunosuppression, which negatively impacts relapse risk and morbidity [8,9,10]. Given these facts, all avenues to govern GVHD must be pursued.

As of a few years ago, HCT has relied on a combination of calcineurin-inhibitor (CNI) plus short course methotrexate (MTX) for GVHD prophylaxis [11,12]. The pair have proven less than fully effective; up to 80% of patients develop GVHD, nearly 30% die from immune-related complications, and many long-term survivors deal with severe forms of chronic-GVHD [8]. The effectiveness of the combination was partially increased at the beginning of the century through the addition of antithymocyte or antilymphocyte globulins (ATG/ATLG) [13,14,15,16].

Based on the results of previous seminal studies demonstrating that cyclophosphamide may target early-proliferating alloreactive T-cells involved in GVHD onset, in 2002, a novel prevention strategy of cyclophosphamide (PTCy) given after bone marrow graft was successfully used as a single-agent GVHD prophylaxis in the haploidentical HCT setting [17,18]. This approach quickly extended to matched related and unrelated donors, and achieved high success in both acute and chronic GVHD control [19,20]. However, preliminary results were limited by graft source, as peripheral blood stem cells (PBSC)—not bone marrow cells—are the worldwide preferred donor source in allo-HCT [6,21]. Using PBSC as a graft—in addition to being the preferred donor option—offers many clinical advantages, e.g., faster engraftment, lower infection risk, and the hope of a more sustained graft-versus-tumor effect. Initial attempts to use PTCy as a sole GVHD prophylaxis after PBSC, however, resulted in a high incidence of GVHD and non-relapse mortality (NRM). Therefore, many groups started evaluating the combination of PTCy with other immunosuppressive agents [22,23,24,25,26]. Tacrolimus/mycophenolate-mofetil (T/MMF)—combining immunosuppression with an immunomodulant effect—was explored in this setting. The revealed results were promising [12,24,26,27].

In light of this, we presented data in 2016 on the first 35 patients we treated with PTCy and tacrolimus/MMF (T/MMF) as GVHD prophylaxis after allo-PBSC-transplant (PBSCT) [26]. The present study updates those published results for 85 patients followed for 36 months, with a focus on immunosuppressive modulation, long-term control of GVHD and late complications, and patient outcomes.

## 2. Materials and Methods

All patients underwent PBSC-based HCT and were matched for HLA-A, B, C, DRB1, and DQB1 alleles to either a related or unrelated donor. The following were deemed acceptable levels of recipient–donor mismatch: an allele–match for HLA-A, B, C, DRB1, and DQB1; a single allele disparity for HLA-A, B, C, or DRB1 or DQB1; two allele disparities for HLA-A, B, or C; a single allele disparity for HLA–DRB1; and a single antigen plus single allele disparity for HLA-A, B, or C. The Supplemental Information sections of the previous publication detailed clinical eligibility and exclusion criteria. All patients signed informed consent before study entry. The study (NCT02300571) was originally conceived by principle investigators as a phase II study; however, it was approved by the Ethics Committee as an observational/interventional study. Our primary objective was to determine the capability of the drug combination to control both acute GVHD (aGVHD) and chronic GVHD (cGVHD), based on cumulative incidence. Secondarily, we sought to measure several key indicators of drug combination success: non-relapse mortality (NRM), infections, overall survival (OS), event-free survival (EFS), cGVHD-EFS, long-term toxicity, and relapse rate. Acute GVHD was diagnosed based on standard criteria, whereas cGVHD was determined by both traditional and NIH criteria [28,29,30]. To account for disease status, stage, and cytogenetic heterogeneity across patients, we also assessed patients using the refined disease risk index [31]. A post hoc analysis based on total immunosuppressive burden associated with the transplantation platform was performed. The analysis considered all posttransplant GVHD control systemic treatments: GVHD or engraftment syndrome, GVHD after donor lymphocyte infusion (DLI) or second alloPBSCT, and GVHD prophylaxis for second alloPBSCT. Aside from this list, death remained the only other immunosuppression risk. Each immunosuppressive agent had the potential to be used at any point throughout the initial and last day of treatment before permanent discontinuation. By exception, when treatment gaps exceeded three months, the discontinuous block durations were summed. Topical agents and budesonide were not included in these analyses. Competing risks for GVHD were graft failure, relapse, DLI, and death.

### 2.1. Conditioning Regimen, Postgraft Immunosuppression, and Supportive Care

Conditioning regimens are reported in Table 1. On day+3 and +4 after transplant, immunosuppression began with intravenous administration of cyclophosphamide (50 mg/kg/day). On day+5 and forward, twice-daily doses of tacrolimus (0.06 mg/kg, targeting trough blood levels of about 5 ng/mL) and thrice-daily doses of MMF (15 mg/kg) were started and provided until day+28, at which point MMF was discontinued. On day+84, a tacrolimus taper was begun. On day+5, a daily G-CSF (5 mcg/kg) was started and continued until the absolute neutrophil count (ANC) >1.0 × 109/L for three consecutive days. As described in the previous publication, patients received prophylaxis for bacterial, fungal, and viral infections, and for *Pneumocystis jirovecii*. In cases of fever (>38.5 °C), blood and urine cultures were collected and wide-spectrum antibiotic intravenous therapy (i.e., piperacillin/tazobactam at 4.5 g q8 h i.v. and vancomycin at 500 mg q6 h i.v.) was started until pathogens were identified or clinical control achieved. Diagnostic and invasive procedures were performed as described in the first report. Standard cytomegalovirus (CMV) monitoring by polymerase-chain-reaction (PCR) was begun on day+10 and continued weekly until day+365 after transplant. Thereafter, monitoring continued according to patients’ follow-up schedule. Treatment with ganciclovir or valganciclovir began when the number of CMV DNA copies rose above 100/mL (unrelated donors) or 500/mL (related donors) for two consecutive measurements, or after a viral load change of >0.5 log IU/mL in peripheral blood plasma. Biweekly plasma samples were taken to detect the Epstein–Barr virus (EBV) up to one year from transplant and then whenever was clinically indicated.

### 2.2. Monitoring after Transplant

Neutrophil engraftment was defined as the first of three consecutive days posttransplant with an Absolute Neutrophil Count ANC of 0.5 × 10^9^/L. Platelet engraftment was defined as a platelet count of 20 × 10^9^/L with no transfusion during the preceding seven days. Posttransplant day+28, +56, +90, +180, and +365 donor chimerism was assessed on circulating myeloid and CD3+ lymphocytes. Chimerism was determined using short tandem repeat (STR)-fragment length analysis (AmpFlSTR^®^Identifiler^®^PCR Amplification Kit, Applied Biosystems), with full chimerism defined as more than 97% donor cells.

### 2.3. Long Term Follow-Up

All patients considered stable (disease in control, off immunosuppressive treatment (IS) and with no signs of GVHD) after HCT were periodically monitored as follow: a transplant team clinic was scheduled every 30–60 days, cardiac function was evaluated at day +100 after HCT (with clinical assessment, electrocardiogram, and echocardiogram), then every 6 months during the first year, and annually thereafter. Pulmonary Function tests (PFT) were performed at day +100 after HCT and then every 6 months during the first year thereafter annually. Thyroid function (TSH, FT3 and FT4) was monitored every 90 days after HCT. Dyslipidemia was monitored at day +100 after HCT and then every six months. During the transplant team clinic, patients were monitored for blood pressure, liver dysfunction, muscle and joint diseases, diabetes, and oral and eye manifestations. Oral and ophthalmologist consults were scheduled annually. The onset of cardiomiopathy with a reduction of ejection fraction (EF) under 50%, as well as valvular or conduction anomalies were considered cardiac complications. Onset of a new obstructive disorder was defined as a reduction of forced expiratory volume in 1 s (FEV1)/forced vital capacity ratio <0.70 (Tiffenau index) in patients with normal Pulmonary function test (PFT). Onset of a new restrictive disorder was defined as reduction in lung volumes with normal Tiffenau index in subjects with normal PFT. Worsening of a preexistent lung disorder was defined as a decrease in pulmonary functions resulting from a previous restrictive or obstructive disorder. When TSH was higher than normal limits and FT4 was lower than the inferior range of normality, it was considered hypothyroidism. Hyperthyroidism was defined as lower than normal TSH with higher than upper normal limits of FT4. Dyslipidemia was diagnosed when total cholesterol was higher than 200 mg/dL, triglycerides were higher than 150 mg/dL or HDL <40 mg/mL [32].

### 2.4. Statistical Analyses

Measures of OS, EFS, and cGVHD-EFS were estimated using the Kaplan–Meier method at their respective 95% confidence intervals (CI) [33,34,35]. A patient death from any cause constituted an OS event, and a relapse or death from any cause was characterized as an EFS event. Chronic GVHD-EFS events, defined broadly per NIH criteria, included any form of cGVHD, relapse, or death. The values for OS, EFS, and cGVHD-EFS were each calculated as the time elapsed between transplant date and event date/censor date, or as the time between transplant date and final follow-up date for patients without an observed event. For patients treated after transplant with DLI, OS and EFS were calculated from the date of first DLI. Kaplan–Meier estimates of DFS, OS, and cGVHD-DFS were compared between groups via log rank statistics and the Cox proportional hazards model. Discontinued immunosuppression time was determined from the date patients ended their immunosuppression drug tapers without subsequent resumption. NRM encompassed all deaths that occurred without evidence of relapse. Standard methods were used to estimate aGVHD and cGVHD rates, relapse or progression, and NRM. Death was treated as a competing risk for all other endpoints. Relapse was treated as a competing risk for NRM. Categorical variables were expressed as proportions and continuous variables were expressed as medians within their respective ranges. Immunosuppressive burden was evaluated considering both reversible and nonreversible transitions between states. Multistate models and the Aalen–Johansen estimator were used to calculate the probability of being: (1) alive and not on immunosuppression, (2) alive and on immunosuppression; or (3) dead (absorbing state). A multistate analysis considering nonreversible transition was also performed to estimate the instantaneous probability of being in one of five states: (1) alive and on immunosuppression, (2) alive and off the first immunosuppression, (3) alive and on the second immunosuppression, (4) alive and off subsequent immunosuppression, or (5) dead [33]. Statistics were performed using IBM-SPSS Statistics v.20, GraphPad-Prism v.5, STATA V.16, R version 3.6.3.

## 3. Results

Eighty-five (85) consecutive patients were enrolled and treated at our Transplant Center between March 2011 and July 2019 (characteristic summary in Table 1). The median follow-up of surviving patients was 36 months (range, 5–107); the median follow-up for the entire population was 26 months.

### 3.1. Engraftment

The median times to neutrophil and platelet recovery were 14 (range, 11–32) and 16 (range, 10–201) days, respectively. Of the 85 patients, 80 (94%) sustained engraftment, 3 (4%) suffered primary graft failure, and 2 (2%) experienced secondary graft failure. Among the primary graft failures, two patients received a second transplant (1 HLA-haploidentical donor, 1 autologous donor), and the third patient died from complications of infection. One of the two (2%) patients with secondary graft failure received a second haploidentical transplant and the other received a CD34+ boost from the original donor. Only one patient experienced delayed engraftment. Median lymphocyte counts (lymphocyte × 10^3^/mmol) were 0.40, 1.23, 1.24, 1.78, and 2.10 at 28, 56, 84, 180, and 365 days after transplant, respectively. On posttransplant day+28, the median donor chimerism for the engrafting patients was >97%, and in not-relapsing patients, chimerism continued at >97% control over time, with no patients requiring transfusion support, even one year after transplant. After transplant, the median time until discharge was 19 (range, 13–174) days. Within the first 100 days after transplant, 16 (19%) patients were readmitted for infection or graft failure and one patient for sinusoidal obstruction syndrome. Across all cases, the complications were treated and the patients were subsequently discharged (Table 2).

### 3.2. Infections

Various bacteria, viruses, and fungal infections affected the study group as described below. Of the 14 (16%) patients who suffered septicemia during engraftment (days 0 to 26), *Escherichia coli* (5 patients), *Pseudomonas aeruginosa* (4 patients) and *Klebsiella pneumoniae* Carbapenemase-producing bacteria (3 patients) were most frequently isolated. *Klebsiella oxytoca* and *Enterococcus faecium* were also isolated in some patients, but less frequently. During the engraftment phase, 16 (19%) patients experienced fever of unknown origin and were treated with empirical antibiotic therapy.

No primary CMV infections were reported, although CMV reactivation (median onset day+37; range, 13–330) was observed in 55 of 85 patients (65%). Preemptive therapy was successful in all cases of CMV. No patients developed CMV reactivation after day+365. No patient developed EBV-related lymphoproliferative disease or an EBV DNA increase requiring anti-CD20 monoclonal antibody administration. Hemorrhagic cystitis due to BK virus was seen in 7 of 85 (8%) patients; however, complete resolution of the infection was achieved in each instance. Two patients suffered HBV reactivation after transplant, but were successfully treated with antiviral therapy. One patient, who was positive for HCV RNA pretransplant, underwent transplantation without hepatic toxicity. This patient began a course of sofosbuvir/velpatasvir on day+100 and complete clearance of the viral load was achieved within three months.

The incidence of proven new invasive fungal infections was 4% one year after transplant. While no patients died from such infections, two experienced *aspergillus* pneumonia and one suffered mucormycosis (Table 2).

### 3.3. Long-Term Toxicity

Cardiovascular disorders also appeared in a number of patients: 5 (8%) patients developed hypertension (median onset day+34), 1 (1%) patient’s ejection fraction fell below 50%, and 1 (1%) patient had a cardiac event during the transplant procedure. After HCT, 16 (32%) patients exhibited PFT changes: new obstructive disorder (12%), new restrictive disorder (10%), or worsened preexisting PFT alteration (10%). Several endocrine system issues emerged in the population: 4 patients (5%) developed thyroid dysfunctions (3 hypothyroidism, 1 hyperthyroidism); 7 patients (15%) became dyslipidemic; 2 (3%) patients emerged with diabetes (Type II) (Table 3). One patient developed oral squamous carcinoma, and two patients developed anterior segment ocular complications (cataracts) that were successfully treated.

No patients developed skeletal complications and no patients developed muscle or joint diseases.

### 3.4. GVHD

All patients were off MMF on day+28, and the median day of tacrolimus discontinuation was +110 (range, 50–333). Immunosuppression had to be restarted for 4 (5%) patients absent disease progression.

**Figure 1:**
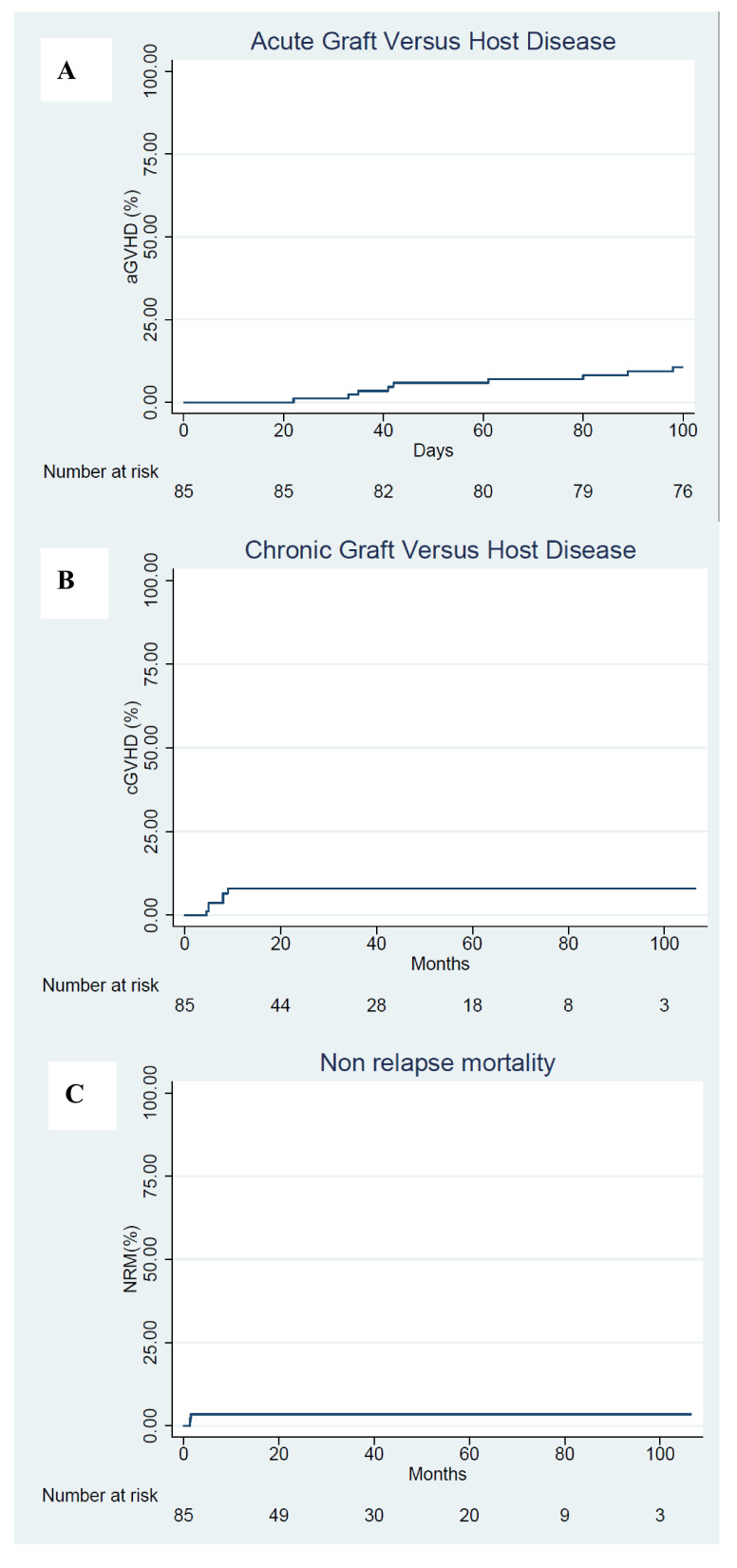
Transplant-related complications. (**A**) Cumulative incidence of acute graft-versus-host disease (aGVHD). (**B**) Cumulative incidence of chronic graft-versus-host disease (cGVHD). (**C**) Non-relapse mortality.

The cumulative incidence across all aGVHD grades was 12% (95% CI; range, 5–19%). Across aGVHD grades II to III, it was 6% (95% CI; 1 to 11%). There were no grade IV cases of aGVHD. The median onset of aGVHD was +52 days (range, 22–99) with no cases of late-onset aGVHD (Figure 1A). All patients with aGVHD were treated with glucocorticoids, for which the median discontinuation was day+136 (range, 30–409).

The cumulative incidence of classical cGVHD was 7% (95% CI; range, 2–13%). Of 6 patients with cGVHD, 2 had limited and 4 had extensive forms. According to NIH-defined criteria, 5 patients had cGVHD: 3 qualified for mild, 1 for moderate, and 1 for severe. The cumulative incidence of NIH-defined cGVHD was 6% (95% CI; range, 2–15%). Overall, the cumulative incidence of patients diagnosed with cGVHD requiring systemic immunosuppressive treatment at one year was 7% (95% CI; range, 2–13%) (Figure 1B). Median time of onset of cGVHD was +193 days (range, 140–268).

All patients with cGVHD were treated with glucocorticoids and a secondary immunosuppressive (IS) treatment (tacrolimus or methotrexate), with all but one discontinued IS at a median of +313 days (range, 215–817). No patient died of GVHD.

**Figure 2:**
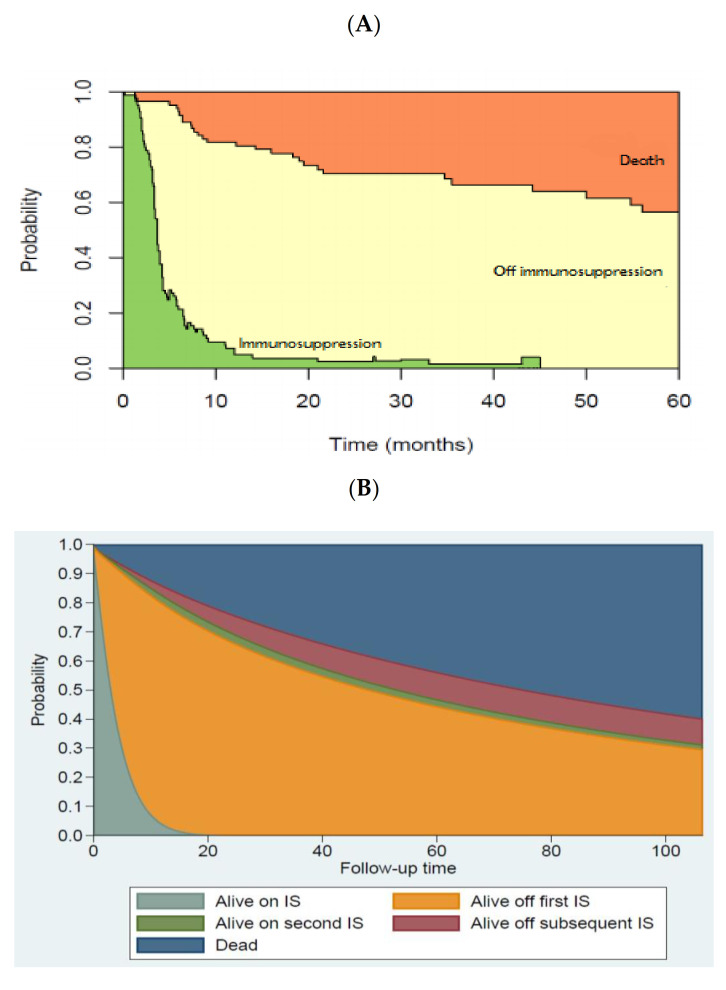
Immunosuppression Burden (**A**) Reversible multistate modeling the instantaneous probability of being in 1 of 3 states: (1) alive, off immunosuppression (off immunosuppression, yellow zone), (2) alive, on immunosuppression (immunosuppression, green zone), or (3) dead (death, orange zone). All patients begin in state 1 on day +5 after transplant, after receiving cyclophosphamide 50 mg/kg on days +3 and +4. Patients may have reversible transition between states (1) and (2) but death was an absorbing state. (**B**) Nonreversible multistate modeling the instantaneous probability of being in 1 of five states: (1) alive, on immunosuppression (IS), (2) alive, off first IS, (3) alive, on second IS, (4) alive, off subsequent IS, or (5) Dead.

Multistate modeling was used to assess the longitudinal immunosuppressive burden. In our cohort of survivors, the probability for surviving patients of being off IS at 12, 24, and 36 months was 92% (75% of total study population), 96% (68% of total study population), and 96% (65% of total study population), respectively. Throughout the follow-up, the off IS state was maintained for all but 5% of patients who required IS restart (Figure 2A,B).

### 3.5. Outcomes

**Figure 3:**
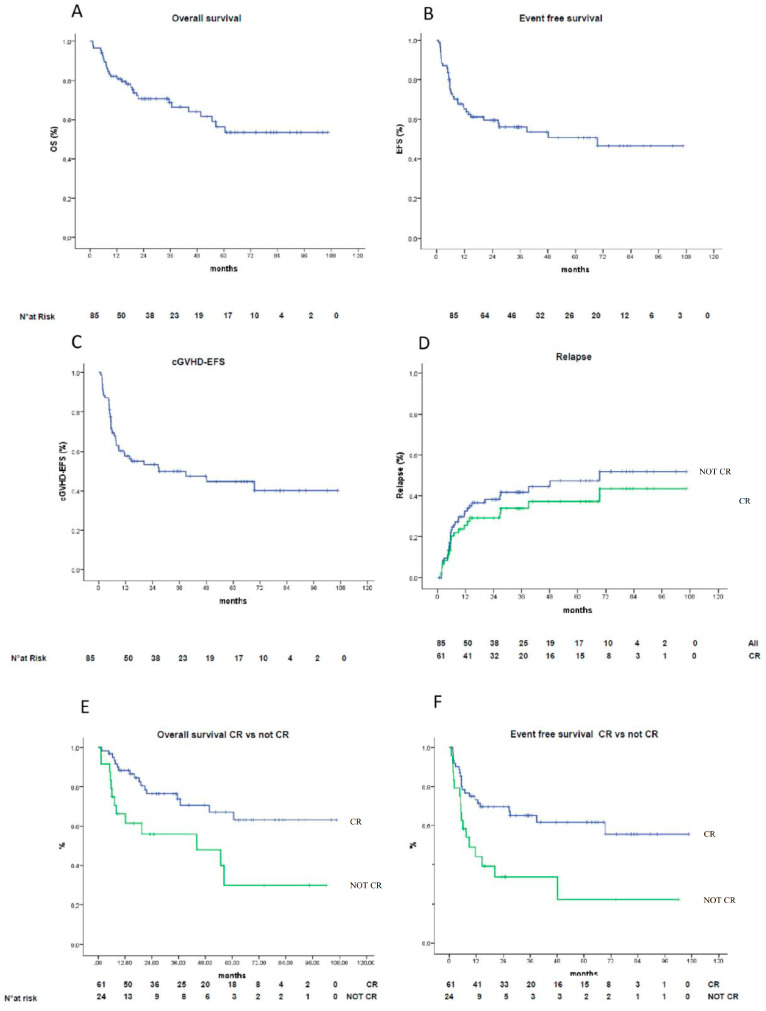
Kaplan–Maier survival curves. (**A**) Overall survival (OS) in the overall population. (**B**) Event-free survival (EFS) in the overall population (**C**) cGVHD-EFS in the overall population, (**D**) Relapse-rate in the overall population (blue line) vs. CR (green line) *p* = 0.336), (**E**) OS stratified by depth of response (CR (blue line) > not CR (green line), *p* = 0.008). (**F**) EFS stratified by depth of response (CR (blue line) > not CR (green line), *p* = 0.003).

The estimated cumulative incidence of NRM at one year was 4% (95% CI; range, 0–7%) (Figure 1C). The one-year EFS and OS values for all patients were estimated as 65% (95% CI; range, 55–75%) and 82% (95% CI; range, 74–90%), respectively. The two-year estimations were 59% (95% CI; range, 51–67%) and 71% (95% CI; range, 62–80%) (Figure 3A,B), respectively. The entire patient cohort has yet to reach the point at which median EFS and OS values can be determined. Rates of cGVHD-EFS at 1-year were estimated as 57% (95% CI; range, 46–68%); at 2-years, 53% (95% CI; range, 45–61%) (Figure 3C). The two-year cumulative incidence of relapse was 38% (95% CI: 26–46%) across all patients, and 30% (95%CI: 18–41%) for patients undergoing HCT in complete response (CR) (Figure 3D). In terms of risk, there was no difference between patients transplanted from siblings compared to those transplanted from matched unrelated donors (MUD) (HR 1.67, 95% CI; range 0.82–3.41). The same holds true for those transplanted from an identical donor compared to those transplanted from a mismatch (HR 0.79, 95% CI; range 0.40–1.56). Most relapses occurred within 8 months. Patients who achieved a first or subsequent CR before transplantation had significantly higher EFS values (73% vs. 43% at 1 year; 69% vs. 32% at 2 years) (Figure 3F) and OS values (88% vs. 65% at 1 year; 77% vs. 55% at 2 years) (Figure 3E), as compared to patients who did not achieve CR.

Among acute myeloid leukemia (AML) patients (33), the cumulative incidence of relapse was 46% overall (95% CI; range, 28–63%), with 34% (95% CI; range, 18–54%) for those transplanted while in phenotypic remission. Among acute lymphoblastic leukemia (ALL) patients, the cumulative incidence of relapse was 50% (95% CI; range, 23–77%), and 42% (95% CI; range, 15–72%) for those in phenotypic remission. Among patients transplanted for lymphoma (17 Non Hodgkin Lymphoma and 3 Hodgkin Lymphoma) the cumulative incidence of relapse was 16% (95% CI; range, 3–40%), but only 1% (95% CI; range, 0,0–44%) in those in complete remission. In multiple myeloma (13 patients), incidence of relapse was 75% (95% CI; range, 42–94%); of note, no patients were transplanted in first CR and almost all patients (9 out of 13) underwent transplants while in stable disease.

Fourteen patients received DLI for disease relapse. Of the 14, 43% (6) were infused from a matched sibling, while the other 57% (8) were infused from an HLA-MUD. The median time between transplant and DLI was 10 months (range, 3–89). The median number of DLI infusions per patient was three (range, 1–13). Seven (50%) patients received systemic disease-specific therapy along with the courses of DLI, while 2 (14%) patients received radiotherapy to focal lesions. Another 2 patients received DLI associated with brentuximab-bendamustine or blinatumomab. Median follow-up after DLI for all patients was 14.7 months (Table 4). The overall response rate (ORR) was 57%, (43CR% and 14PR%) but in a further 3 patients (21%), disease control was achieved. After DLI treatment, the incidence of aGVHD (grades I–II) was 31% with no grade III–IV cases. Only 1 (7%) patient developed cGVHD. All patients with GVHD received a short course of systemic immunosuppression treatment. Across all patients who received DLI, none died from an adverse DLI event. The estimated rates of 1-year EFS and 1-year OS from the first DLI were 52% (95% CI; range 26–78%) and 71% (95% CI; range, 47–95%), respectively.

## 4. Discussion

It is likely that the treatment landscape of many hematological malignancies will profoundly change in the coming years due to the introduction of sophisticated cell therapies like chimeric antigen receptor (CAR) T cells [1,36,37,38,39,40].

Right now, allogeneic HCT remains the only potentially curative approach able to handle the clonal heterogeneity of the disease and able to be scaled to all eligible patients [41,42,43]. Hence, any efforts to improve the procedure’s safety and effectiveness is highly relevant to the clinical community. This study with more patients and a longer follow-up period confirmed the extreme benefit offered by combination PTCy/T/MMF after allogeneic PBSC-HCT in controlling major transplant complications and maintaining transplant-related mortality below 4%.

These results present several clinical benefits and development opportunities worthy of consideration. Clinical benefits of the therapy include: low incidences of any GVHD forms, limited need for steroid therapy or other IS forms, and high proportions of patients who discontinue IS early and definitely. Here, the cumulative incidence of aGVHD (12% with no grade IV cases) and cGVHD (7% with no extensive cases), regardless of donor type or degree of HLA mismatching, compared quite favorably with standard CNI-based prophylaxis, for which aGVHD rates are 60–80%, and cGVHD rates are 30–60% [5,8,11,12,21]. Although promising, new strategies appear to have less-pronounced activity levels. These strategies include: PTCy after BMT-HCT (39–60% aGVHD and 6–21% cGVHD), ATG-based regimens (23–56% aGVHD, and 16–31% cGVHD), and T/MMF/Sirolimus regimens (26% aGVHD and 49% cGVHD) [13,14,15,16,18,19,20,33,44,45].

In the present study, most patients were freed from IS after a median of 4 months. This condition persisted over the long term to become 92% probable for surviving patients (75% of the entire patient cohort) at 12 months and 96% probable at 24 and 36 months, respectively (68% and 65%, respectively, of the entire population). In traditional calcineurin-based regimens, it is uncommon to discontinue IS (20%) and relatively common for other IS forms (including steroids) to be used for long periods after transplantation. Additionally, in newer strategies, the burden of IS persists— albeit significantly less pronounced [25,33,46,47]. After PTCy-BMT, the cumulative incidence of steroid use at three years ranges between 46& and 68%; the probability of being alive and free from IS ranges between 48–56%, with 10–20% of patients conditioned with busulfan/fludarabine still on IS at 3 years [33]. In ATG regimens, grade II–IV aGVHD requires additional IS in one-third of patients, and the rate of cGVHD at two years is approximately 30%, plateauing between 10 and 30% for periods beyond two years [48,49]. In MUD transplants, the probability of being alive and off IS lies between 50% and 55%, regardless of time (12, 24, or 36 months) [49].

Recently, three studies reported on the activity of the PTCy/T/MMF approach. The first was a retrospective European Bone Marrow Transplantation (EBMT) study in MUD HLA-mismatched patients. The second represented the first phase III trial between the two strategies (PTCy/T/MMF and conventional immunosuppression). The third was a Blood and Marrow Transplant Clinical Trials Network (BMT/CTN) phase II study in which PTCy/T/MMF was compared with other two experimental regimens, adopting a cohort of patients treated with tacrolimus and methotrexate (T/MTX) as control for all the three experimental arms. Overall, their results demonstrated that PTCy had the best profile for controlling GVHD and immunosuppression length [50,51,52]. The present update, with the most extended follow-up period, augments the data of these three large studies, enriching them with additional detail on immunosuppression regimen modulation over time—likely a foundational element for future development of the strategy.

Undoubtedly, another set of benefits that GVHD protection provided was evidenced by the low incidence rates of late infections (LI) in our patients. A large retrospective study by the Center for International Blood and Marrow Transplant Research reported that approximately 21% of the deaths that occur two years post-transplant result from LI. To date, none of the patients in our study who survived more than 24 months has died of a cause other than disease relapse [53,54]. This result distinguishes the PTCy strategy from classical CNI/MTX or CNI/MTX/ATG-ATLG regimens, considering the burden of serious LI present in both of them even long after the date of transplant [11,12,48,49,55].

Control of GVHD, limited IS duration, and low LI rates all contribute to the low long-term organ toxicity rates reported here [56,57,58,59]. Among our surviving and non-relapsing patients, the onset of cardiovascular, respiratory, renal, or metabolic disease has been rare. Indeed, a good proportion of our patients resumed their occupations between 9 and 12 months after transplant (verified by personal communication). This is the first time that inpatients treated with PTCy have reported a descriptive snapshot regarding late organ toxicity; while it is not possible to draw a comparison with other studies with PTCy and a longer follow up is needed, the presented data compares well with those registered in large registry cohorts [32].

Despite the major clinical achievements described here, disease relapse was the principal cause of treatment failure with PTCy/T/MMF. Compared to other GVHD prevention methods, it was not associated with a higher risk of relapse (PTCy-BMT 22–44% ATG based regimens 11–42%) [13,14,15,16,18,19,20]. The fact that most relapses were concentrated in the first 8 months, however, suggests that the balance between the search for tolerance and trigger of graft versus tumour has to be better modulated. Two possible strategies may be pursued: first, in patients affected by a high-risk malignancy, implementing a faster IS taper to be free from IS at day +60; second, consider the robust and quick immunological tolerance that PTCy/T/MMF often invokes and insert donor lymphocyte infusions (DLI) as part of the strategy [60,61]. In this regard, the results and toxicity achieved with DLIs paves the way for evaluation of their use early after transplant and for consideration of PTCy/T/MMF as a basis from which to develop more selective forms of adoptive cell therapy.

While it is beyond the scope of this study of GVHD prevention to comment on the outcomes (OS, EFS), their median values demonstrated a satisfactory temporal trend and are worth noting. Moreover, GVHD-EFS (53% at 2 years) and NRM (4%) portend potential activity and safety benefits. They may also be beneficial when considering allo-HCT for diseases with strong evidence of graft-versus-tumor effects, but for which procedure toxicity and novel forms of therapy have halted further investigations [62,63,64].

Last, the present study has methodological and translational limitations that cannot be ignored, and which preclude definitive conclusions. Notwithstanding, it offers solid data for future clinical trials and reinforces the philosophical transformation of allogeneic HCT from chemo/radiotherapy-based approaches to more immunologically-safe platforms for the cure of hematological malignancies.

## 5. Conclusions

The present study provides evidence that PTCy/T/MMF after allo-PBSCT allows a fast engraftment, reduces GVHD substantially, and releases most patients from IS early and definitely. Those factors not only positively impact LI and long-term posttransplant complications, but do not appear to obstruct the onset of a sustained graft-versus-tumor effect. Post-transplant relapses continue to represent a pitfall of the strategy. The limited NRM described is noteworthy, and may contribute to continuing explorations of allogeneic HCT as an effective cell-based therapy for the cure of hematological malignancies.

## Figures and Tables

**Table 1:** Patient and Donor Characteristics. AML: acute myeloid leukemia; ALL: acute lymphoblastic leukemia; MDS: myelodisplastic syndrome; BMT: bone marrow transplantation; CR: complete response; MDR: minimal residual disease; TBI: total body irradiation; MMF: mofetil mycophenolate; CMV (Cytomegalovirus); D (donor); R (recipient). Disease risk corresponding to Center for International Blood and Marrow Transplant Research CIBMTR classification. * Two patients had an antigen disparity at DQA1; Cyclophosphamide was also given before alloPBSCT at 14.5 mg/kg on two consecutive days. ° Cyclophosphamide was also given before PBSCT at 10 mg/kg on two consecutive days. CD 34+ cell doses of cell were available for all patients; CD3+ doses only for 89% of patients.

Patients’ and Donors’ Characteristics	Total (N = 85)
**Age at transplant (years)**	
Median	51 y
Range	19–74
**Sex**	
Male	51 (60%)
Female	34 (40%)
**Disease**	
**AML**	**33 (39%)**
AML	25 (75%)
Relapsed AML	8 (25%)
**ALL**	**14 (16%)**
ALL	6 (43%)
Relapsed ALL	8 (57%)
**Non-Hodgkin Lymphoma**	**17 (20%)**
**Multiple Myeloma**	**12 (14%)**
**MDS**	**4 (5%)**
**Hodgkin Lymphoma**	**3 (4%)**
**Aplastic Anemia**	**1 (1%)**
**Myelofibrosis**	**1 (1%)**
**Disease status at Transplant**	
1° CR	40 (47%)
>1° CR	22 (26%)
Active disease	23 (27%)
**CIBMTR risk group**	
**Very High**	6 (7%)
**High**	29 (34%)
**Intermediate**	32 (38%)
**Low**	17 (20%)
**Not applicable**	1 (1%)
**Source of stem cell**	
peripheral blood stem cell	85 (100%)
**Sex mismatch**	
No	47 (55%)
Yes	38 (45%)
Female into male	20 (24%)
**Donor age, years**	
Median	29 y
Range	16–68
**Source of graft**	
sibling	20 (24%)
unrelated	65 (76%)
**HLA match**	
10/10	47 (55%)
9/10	23 (27%)
8 */10	15 (18%)
**CMV serology**	
CMV D−R−	2 (2%)
CMV D+R−	31 (37%)
CMV D−R+	2 (2%)
CMV D+R+	50 (59%)
**Conditioning regimen**	
Busulfan + Cyclophosphamide	25 (30%)
Thiotepa + Treosulfan	11 (13%)
Fludarabine + Treosulfan + Thiotepa	7 (8%)
Treosulfan + Fludarabine + Cyclophosphamide	5 (6%)
Treosulfan + Cyclophosphamide	11 (13%)
Melphalan + Cyclophosphamide	5 (6%)
Treosulfan + Cyclophosphamide + TBI 2Gy °	4 (5%)
Melphalan + Cyclophosphamide + TBI 2Gy °	4 (5%)
Busulfane + Fludarabine	5 (6%)
Fludarabine + Melphalan + TBI 2Gy °	3 (2%)
Fludarabine + Thiotepa + Cyclophosphamide	4 (5%)
Cyclophosphamide + ATG + Fludarabine	1 (1%)
Infused cell dose * CD34+ cell × 10^6^/kg,	
Median	7 (range 2–15)
**CD3+ cell × 10^8^ kg**	
Median	2.89 (range 1.123–10.372)
**Total Nucleated Cells × 10^8^/kg**	
Median	12.1 (range 6.9–15.739)

**Table 2:** Post-transplant data. ° Peripheral blood lymphocyte count was available on day 28, 56, +84, +180, and +365 for all survivor patients. § Chimerism on peripheral blood was available for all patients alive without disease relapse. † Toxicities were graded according to standard National Cancer Institute Common Terminology Criteria for Adverse Events version 4.0. ‡ Hemorrhagic cystitis and cerebral hemorrhage.

Post-Transplant Data (*n* = 85)	
**Engraftment median time**	
Neutrophils engraftment > 0.5 × 10^9^/L	14 days (range 11–32)
Platelets engraftment > 20 × 10^9^/L	16 days (range 10–201)
**Peripheral Blood Lymphocyte count** °	
**Day+28** Median (U/µL)	400 (range 10–3640)
**Day+56** Median (U/µL)	1200 (range 250–5000)
**Day+84** Median (U/µL)	1200 (range 360–5000)
**Day+180** Median (U/µL)	1800 (range 400–4900)
**Day+365** Median (U/µL)	2100 (range 110–5600)
**Chimerism** §	
**Day+28**	>97% of patients alive and not relapsed
**Day+56**	>97% of patients alive and not relapsed
**Day+84, +180, +365**	>97% of patients alive and not relapsed
**CMV reactivation**	
Incidence	55 (65%)
Median day of reactivation	37 (range 13–330)
**Bloodstream infection during engraftment**	
**(day 0–26)**	
Incidence	14 (16%)
**Sort of microorganism**	
*E. Coli*	5 (36%)
*Pseudomonas aeruginosa*	4 (29%)
*Klebsiella pneumoniae* carbapenemase-producing	3 (21%)
*Klebsiella Oxytoca*	1 (7%)
*Enterococcus Faecium*	1 (7%)
**Invasive Fungal infection at 1 year**	
Incidence	3 (4%)
**Toxicity (G3–G4)** †	
Mucositis	16 (19%)
Hemorrhage ‡	6 (7%)
Liver enzymes elevation	5 (6%)
Sinusoidal obstruction disease (SOS)	4 (5%)
Hypocalcemia	1 (1%)
Hyperbilirubinemia	1 (1%)

**Table 3:** Long Term Toxicities. PFT = Pulmonary Function Test; ° data were available for 51 patients; ^x^ Data were available for 77 patients; ^a^ Data were available for 47 patients; ^b^ Data were available for 76 patients; ^c^ Data were available for 63 patients; ^d^ Data were available for 80 patients, defined as ejection fraction < 50%.

Long Term Toxicities (All Data Reflect Median Follow Up of 36 Months)	
**Modification of PFT °:**	
Global Incidence	16 (32%)
New obstructive disorder	6 (12%)
New restrictive disorder	5 (10%)
Worsening of a preexistent disorder	5 (10%)
**Emergence of Thyroid disfunction** ^x^	4 (5%)
Hypothyroidism	3 (4%)
Hyperthyroidism	1 (1%)
**Emergence of Dyslipidemia** ^a^	
Global incidence	7 (15%)
**Emergence of Diabetes** ^b^	
Global incidence	2 (3%)
**Cardiovascular disorders**	
**Emergence of Hypertension** ^c^	
Global incidence	5 (8%)
**Emergence of Hypokinetic Cardiomyopathy** ^d^	
Global incidence	1 (1%)

**Table 4:** Donor Lymphocyte infusions indications and outcomes.

DLI (*n* = 14 Patients) *	
**Indication for treatment**	
disease relapse	14 (100%)
**Source of DLI**	
matched sibling	6 (43%)
HLA-matched unrelated donors	8 (57%)
**Median time between transplant and DLI** **Median number of DLI infusions = 3**	10 months (range 3–89) 3 months (range 1–13)
**Overall response rate**	57%
**Disease control rate**	78%
**Incidence of acute GVHD grade I–II**	33%
**Incidence of acute GVHD grade III–IV**	0%
**Estimated 1-year EFS**	52% (95% CI, 26–78%)
**Estimated 1-year OS**	71% (95% CI, 47–95%)

* Median follow-up post- donor lymphocyte infusions (DLI) for all patients was 14.7 months.

## Data Availability

The data presented in this study are available on request from the corresponding author.
